# *Babesia microti* Causing Intravascular Hemolysis in Immunocompetent Child, China

**DOI:** 10.3201/eid2903.220888

**Published:** 2023-03

**Authors:** Jiafeng Yao, Guoging Liu, Yang Zou, Jin Jiang, Shaogang Li, Heng Wang, Xiaoling Cheng, Rui Zhang, Kaige Zhang, Chunyan Wei, Runhui Wu

**Affiliations:** Beijing Children’s Hospital, Beijing, China (J. Yao, G. Liu, J. Jiang, X. Cheng, R. Zhang, K. Zhang, R. Wu);; Capital Medical University, Beijing (Y. Zou, S. Li);; Peking Union Medical College School of Basic Medicine, Beijing (H. Wang, C. Wei)

**Keywords:** Babesia microti, parasites, piroplasm, infection, severe intravascular hemolysis, immunocompetent child, preschooler, China

## Abstract

We report a case of *Babesia microti* infection in an immunocompetent child <5 years of age that caused fever and severe intravascular hemolysis. Physicians in China should be aware of babesiosis, especially in the differential diagnosis of immune hemolytic anemia with negative results for antiglobulin tests.

Babesiosis, caused by tickborne zoonotic protozoa of the genus *Babesia*, is an emerging health risk to humans. Among the known *Babesia* species infecting humans, *B. microti* is the most common cause of human babesiosis (*1*). In China, *B. microti* has caused >100 human cases of babesiosis (*2*), but nearly all have been reported in adults, particularly the elderly.

*B. microti* babesiosis has rarely been reported in immunocompetent children in China. We reported a case of severe intravascular hemolysis caused by *B. microti* infection in an immunocompetent preschooler from Shandong Province, China.

The patient, a girl 4 years and 9 months of age, had fever develop (highest temperature 39°C) on September 30, 2021. Antimicrobial drug treatment was not effective. Four days later, her urine became dark, and she had abdominal pain. On October 9, 2021, she was admitted to the hospital because of severe anemia and abnormal laboratory test results (Table). She had shock after a discharge of dark brown urine.

**Table:** Laboratory test results for *Babesia microti* causing intravascular hemolysis in immunocompetent child, China*

Variable	Age-adjusted reference value or range	Value
Epstein-Barr virus	Negative	Negative
*Mycoplasma pneumoniae*	Negative	Positive
IgM titer	<1:80	1:80
Drug-resistance gene	Negative	Negative
Leukocyte count, × 10^9^ cells/L	4.9–12.7	4.36
Erythrocyte count, × 10^12^ cells/L	4.1–5.5	2
Hemoglobin, g/L	115–150	57
Mean corpuscular volume, μm^3^	76–88	83
Mean corpuscular hemoglobin concentration, g/L	309–359	343
Platelet count, × 10^9^/L	187–475	100
% Reticulocytes	0.5%–2.5%	2.07%
Clotting function	NA	Normal
Bilirubin, μmol/L		
Total	3.42–20.5	55.98
Direct	0–3.42	13.76
Indirect	0–17.1	42.22
Aspartate aminotransferase, U/L	14–44	196.2
Alanine aminotransferase, U/L	7–30	187.6
Lactate dehydrogenase, U/L	110–295	2,899
Antiglobulin tests		
IgG	Negative	Negative
C3d	Negative	Negative
DAT	Negative	Negative
Control	Negative	negative
Spot urine sample		
Urine color	Light yellow	Brown
Ketone body	Negative	1+
Protein	Negative	2+
Urinary bilirubin	Negative	1+
Occult blood	Negative	3+
Centrifugal microscopy for erythrocytes, HPF	0–3	0
Centrifugal microscopy for leukocytes, HPF	0–3	0
Chest computed tomography	NA	Few shadows in lower lobe of right lung, no exudative lesion
Abdominal ultrasound	NA	Splenomegaly

*DAT, direct antiglobulin test; HPF, high-power field; NA, not applicable.

To stabilize her vital signs, we began repeated blood transfusion for supportive treatment. Azithromycin and immune regulatory treatment (high-dose methylprednisolone, 10 mg/kg/d for 3 days, and intravenous immunoglobulin, 1 g/kg/d for 2 d) were not effective. Her symptoms worsened, and her hemoglobin level remained at <60 g/L (Appendix Figure, panel A). On the basis of those findings, we excluded congenital hemolytic anemia and autoimmune hemolytic anemia.

We examined her blood smear and observed parasites in the erythrocytes (Appendix Figure, panel B). We used a genus-specific 18S rRNA PCR described previously (*3*) to confirm *Babesia* infection by amplification of a 515-bp fragment (Appendix Figure, panel C). Test results for malaria infection was negative. Subsequent sequencing of the fragment and BLAST analysis (https://blast.ncbi.nlm.nih.gov/Blast.cgi) of the nucleotide sequence showed 100% similarity with *B. microti* RI strain. Those results confirmed the diagnosis as a *B. microti* infection causing severe intravascular hemolytic anemia.

The girl’s parents recalled that the child had been in a wild chestnut forest in a suburb of Zaozhuang City, Shandong Province, on September 14, 2021. They found ≈20 red papules and an itching sensation on the trunk and limbs. The papules subsided within 3 days. There were no other complications of babesiosis, such as splenic infarction, acute respiratory distress syndrome, or disseminated intravascular coagulation.

The child was given atovaquone and azithromycin (*4*) for 21 days, and the urine color became clear within 24 hours. The frequency of erythrocyte transfusion was reduced gradually, and the hemolysis was controlled (Appendix Figure, panel A). On the 10th day after the treatment began, molecular detection of *B. microti* showed a negative result. The child has been monitored for >6 months and is in good health. 

In this case, we confirmed the severe intravascular hemolysis caused by *B. microti* infection in an immunocompetent child <5 years of age. For children in China, although babesiosis caused by *B. venatorum* and *B. crassa*‒like parasites was detected in epidemiologic surveys (*5*,*6*), few children who have babesiosis caused by *B. microti* and severe hemolysis have been reported. Thus, babesiosis is still unfamiliar to pediatricians. This case implied that *Babesia* infection might be underdiagnosed in China. It is imperative for pediatricians and clinicians to be aware of babesiosis, which has become a newly emerging public health threat globally.

In Shandong Province, where this child lived, only 2 adults who had babesiosis and severe anemia caused by *B. divergence* were documented (*7*), but *B. microti*‒positive (0.58%) hard ticks from the Jiaodong Peninsula in Shandong Province were reported (*8*). Given that the child did not have a splenectomy, take immunosuppressive drugs, receive previous blood transfusions, or have other travel histories, her travel history to the wild chestnut forest and subsequent red, itching papules provided strong evidence for *B. microti* infection by tick bites. Detailed epidemiologic survey of *Babesia* infection in tick vectors and reservoir animals in local areas is necessary to provide guidelines for better prevention and control of babesiosis.

Severe babesiosis in immunocompetent persons <50 years of age is rare; only 2 cases have been reported (*9**,**10*). We report a case of *B. microti* infection causing severe illness in an immunocompetent child. Better understanding of the pathogenesis of this parasite is necessary. This case indicates that babesiosis cannot be ignored in severe intravascular hemolysis. For patients who have intermittent fever and intravascular hemolysis but negative results for antiglobulin tests, babesiosis should be considered in the differential diagnosis, especially in areas where the tick vector is present. A timely and appropriate treatment for patients who have severe disease that is recognized early is needed.

Appendix Additional information on *Babesia microti* causing intravascular hemolysis in immunocompetent child, China.
