# Supplementary material for: Babesia microti Causing Intravascular Hemolysis in Immunocompetent Child, China
Source: Emerg Infect Dis. 2023 Mar;29(3):667–9. doi: 10.3201/eid2903.220888 (PMC9973695; doi:10.3201/eid2903.220888)
Supplement: Appendix — Additional information on Babesia microti causing intravascular hemolysis in immunocompetent child, China. [file 22-0888-Techapp-s1.pdf]

Article DOI: https://doi.org/10.3201/eid2903.220888

Babesia microti Causing Intravascular Hemolysis in Immunocompetent Child, China

Appendix

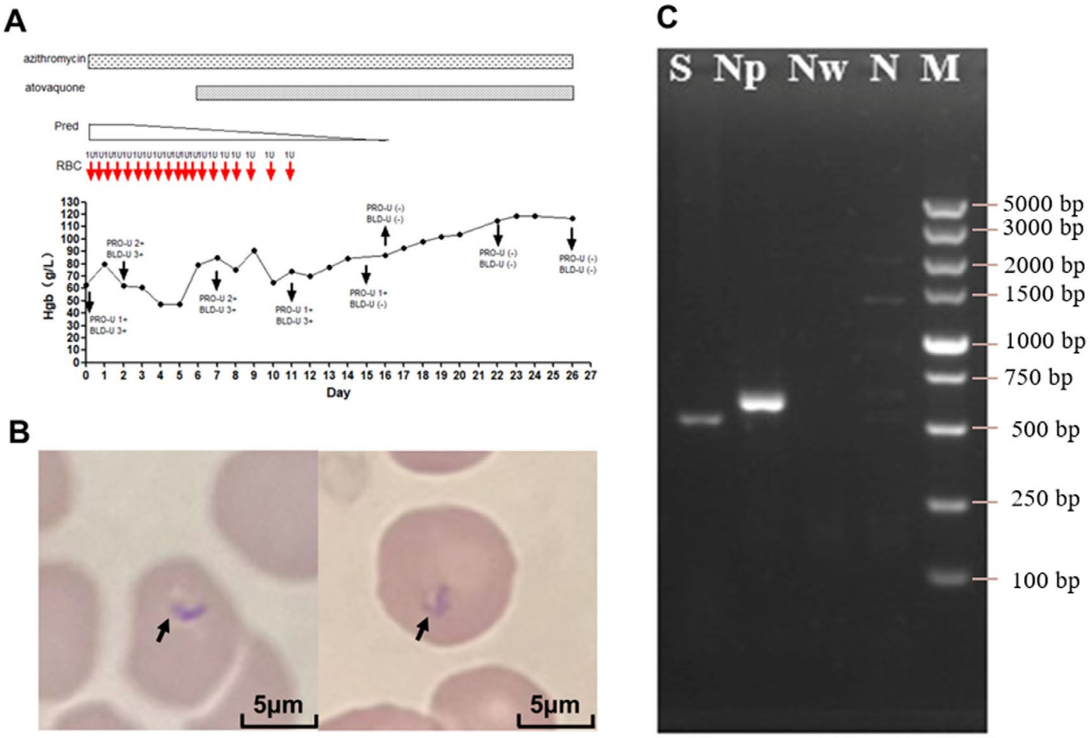

Appendix Figure. *Babesia microti* infection causing severe intravascular hemolysis in an immunocompetent child, China. A) Time course of infection. BLD-U, urine occult blood; Hgb, hemoglobin; Pred, prednisolone; PRO-U, urine protein RBC, red blood cell. Each red arrow indicates transfusion of 1 unit of RBCs. B) Blood smear showing parasites (arrows) in RBCs (a strain with 100% similarity with *B. microti* strain RI; hematoxylin and eosin stained). C) Electrophoresis of genus-specific 18S rRNA PCR products showing *Babesia* spp. infection (intense amplification of a 515-bp fragment) in patient. Lane S, *Plasmodium falciparum* 3D7 strain; lane NP, RBCs from patient; lane Nw, water; lane N, standard RBCs; lane M, DNA marker.
